# Predation of Ladybird Beetles (Coleoptera: Coccinellidae) by Amphibians

**DOI:** 10.3390/insects3030653

**Published:** 2012-07-18

**Authors:** John J. Sloggett

**Keywords:** alkaloid, Anura, aposematism, Caudata, chemical defense, chemical resistance, Coccinellidae, foraging cues, generalist predation, prey discrimination

## Abstract

Studies of predation of ladybird beetles (Coccinellidae) have focused on a limited number of predator taxa, such as birds and ants, while other potential predators have received limited attention. I here consider amphibians as predators of ladybirds. Published amphibian gut analyses show that ladybirds are quite often eaten by frogs and toads (Anura), with recorded frequencies reaching up to 15% of dietary items. Salamanders (Caudata) eat ladybirds less frequently, probably as their habits less often bring them into contact with the beetles. Amphibians do not appear to be deleteriously affected by the potentially toxic alkaloids that ladybirds possess. Amphibians, especially frogs and toads, use primarily prey movement as a release cue to attack their food; it is thus likely that their ability to discriminate against ladybirds and other chemically defended prey is limited. Because of this poor discriminatory power, amphibians have apparently evolved non-specific resistance to prey defensive chemicals, including ladybird alkaloids. Although amphibian-related ladybird mortality is limited, in certain habitats it could outweigh mortality from more frequently studied predators, notably birds. The gut analyses from the herpetological literature used in this study, suggest that in studying predation of insects, entomologists should consider specialized literature on other animal groups.

## 1. Introduction

Ladybird (coccinellid) beetles have a prodigious array of natural enemies. In the last two decades there has been intense interest in the parasites and pathogens of ladybirds [1,2,3,4] and in intraguild predation by and of ladybirds [5,6,7,8]. However predation of ladybirds by more generalist predators has remained largely overlooked. 

A possible reason for the limited amount of attention devoted to such predation might be the formidable chemical defenses of ladybirds, which are often assumed to deter generalist predators. These are based on interspecifically variable distasteful or toxic alkaloids that are present throughout all ladybird life history stages [9,10,11,12]. Additionally, methylalkylpyrazines provide ladybirds with an unpleasant smell, which serves as an olfactory equivalent of warning coloration [13]. Although these chemical defenses clearly do provide ladybirds with a degree of protection from predation, as might be expected (e.g., [9,14,15,16,17]), this is by no means universal. Exceptions are numerous and informative, indicating a particular foraging situation where a predator is prepared to accept prey that is of low quality or even risky to consume, or, alternatively, some form of evolved immunity to some or all of these defensive chemicals (e.g., [18,19,20]). 

Past studies of generalist predation have tended to focus on birds, in large part because of the role they apparently play in the evolution of ladybird color patterns (e.g., [15,17,18]), and ants, which are more often competitors of ladybirds than predators [16,21,22]. Nonetheless predation is not limited to these groups. Ceryngier *et al.* [23] state that “Predation by vertebrates concerns virtually all the main groups: fish, amphibians, reptiles, birds and mammals”. This argues that vertebrate groups other than birds should be examined in more detail. 

In this paper, I consider the extent of predation of ladybirds across the three amphibian orders, in particular focusing on an extensive but, by entomologists, vastly underutilized resource, the very numerous studies of amphibian gut contents that have been carried out since the 1800s. I then go on to discuss observations relating to the effects of ladybird defensive chemistry on amphibians. I conclude by considering why amphibians eat ladybirds and why they are not more strongly affected by ladybird chemical defenses when they do. 

## 2. Gut Analyses of Amphibians

Since the 19th century, amphibians have been the subject of frequent dietary analyses, particularly using gut dissection (e.g., [24,25,26,27]) and, more recently, also the less destructive stomach-flushing methodology [28,29]. Although noted as of value to entomologists in the past (e.g., [30,31]), this data seems rarely to be used by entomologists now. There are probably a number of reasons for this, the most important being a lack of awareness that so much data exists. This is exacerbated by the fact that much of this information is published in relatively obscure journals, even today, and that much more of it is many decades old. It should be borne in mind that, because of the latter two factors, even this review cannot be fully comprehensive, and that for every reference discussed, others exist that have not been seen by the author.

In order to obtain a relatively unbiased comparison of the extent of predation of ladybirds by the three orders, Anura (frogs and toads), Caudata (salamanders and newts) and Gymnophiona (caecilians), I collected papers on gut analyses of amphibians from a 10-year period (2002–2011) using the Web of Science database and Google as starting points for searches. I excluded papers on specialized feeding (e.g., [32]) and exclusively aquatic species (e.g., [33]). Papers included in the analysis were only those that identified beetles and other prey to the family level, a prerequisite for the identification of ladybirds (members of the Coccinellidae) in the diet. 

A total of 21 papers from 2002–2011 detailing gut analyses of 28 species of frogs and toads were examined. Seven of the 21 papers (33%) included examples of coccinellid predation, with predators of ladybirds comprising six of the 28 species (21%). Only seven papers on newts and salamanders, each covering one different species, were found, and of these only one (14%) included a record of predation of coccinellids. There is very limited available work on the diet of caecilians [34]. Of the five papers reviewed, two, each covering one species, included no Coleoptera as prey at all. In the other three covering a total of four species, although beetles formed a part of the diet, they were generally infrequent (0.7%–5.9% of all dietary items). In most cases no indication of beetle identity was given, and where it was, they were predominantly soil-dwelling larval stages [35].

In the comparison of the amphibian orders, frogs and toads appear to most frequently include ladybirds in their diet. It seems likely that this is a reflection of the habitats they live in and possibly their jumping ability. Ladybirds are active in vegetation off the ground, although they may hibernate or aestivate at ground level. It is when they are active that they are moving and most vulnerable to anuran attack (see Section 4.1). Frogs and toads often live in open vegetated areas, even actually in the vegetation (for example treefrogs), while newts and salamanders tend to live on the ground, in damper areas, such as forest floors, caves and ponds, and those caecilians that are not aquatic live underground.

### 2.1. Anuran Predation of Ladybirds

The conclusion above, that of the amphibian orders anurans most frequently include ladybirds in their diets, appears to be borne out by the very abundant records of anuran predation of coccinellids, including by members [36] of the Bombinatoridae [37], Scaphiopodidae [38,39], Pelobatidae [40], Hylidae [41,42,43,44,45,46,47], Leptodactylidae [48] and Hyperoliidae [49], as well as very abundant records from the Bufonidae [25,30,50,51,52,53,54,55,56,57] and Ranidae [26,40,51,58,59,60,61,62,63,64,65,66,67,68,69,70]. The particular preponderance of records for ranids (true frogs), bufonids (true toads) and to a letter extent hylids (treefrogs) probably reflects the rather more abundant work on species in these groups, due to their common and widespread occurrence in Europe and North America. 

In some cases records are of one or a few isolated ladybirds in a diverse diet (e.g., [39,48,51,63]). From three related studies of the yellow bellied toad, *Bombina variegata* (L.), including 13 separate samplings of stomachs [37,71,72], there is only one example of ladybird predation [37]. It has been suggested that such low numbers are a consequence of avoidance or rejection of unpalatable or warningly colored prey [42,43,49]. However such low levels of predation are by no means universal. In the toad *Anaxyrus fowleri* (Hinckley) (=*Bufo woodhousei fowleri* Hinckley), 93 out of 497 stomachs (18.7%) collected over a season were found to contain the remains of coccinellids [54]. Similarly 127 coccinellid adults and 532 larvae were recovered from 296 stomachs of American spadefoot toads, *Spea hammondii* (Baird) (=*Scaphiopus hammondi* Baird): in total ladybirds comprised 12.5% of the dietary items recorded [38]. A number of gut analyses have recently been carried out on the frog *Pelophylax ridibundus* (Pallas) (=*Rana ridibunda* Pallas) in Romania, Bulgaria, Russia and Turkey, [66,67,68,70,73]: of 10 separately tabulated samplings of stomachs in these papers, only three do not include coccinellids. The proportion of coccinellids in the diet of *P. ridibundus* can reach almost 7% [68]. Other members of the *Pelophylax esculentus* species complex may also prey on ladybirds [40]; in general beetle predation by members of this group seems to be high, with beetles comprising about a fifth of dietary items [74]. 

There is some reason to believe that many species take coccinellids in proportion to their occurrence in the habitat. Two different studies of phylogenetically separated anuran species, the spring peeper, *Pseudacris* (=*Hyla*) *crucifer* (Wied-Neuwied), (Hylidae) and the northern leopard frog, *Lithobates pipiens* (Schreber) (=*Rana pipiens pipiens* Schreber), (Ranidae), both in Ithaca, New York, recorded high numbers of coccinellids in their diets in 1962 [44,62]. Coccinellids reached 15% of items in the diet of juvenile *P. crucifer* in June [44] and 11% of the diet of juvenile *L. pipiens* [62]. Proportions in the diets of adults were much lower. An explanation consistent with these simultaneous and co-occurring high dietary ladybird abundances would be a population explosion of ladybirds occurring in this year: this is not an uncommon phenomenon in aphid-eating Coccinellidae in summer after breeding [75]. A ladybird population explosion would also explain the higher ladybird abundances in the diets of juveniles than adult frogs. The adults were apparently collected over a longer period than the juveniles: the latter would have been largely collected after metamorphosis in summer, at the time when a ladybird population explosion would be occurring. Thus the diet of juveniles would more strongly reflect a sudden summer increase in ladybird numbers. 

Unfortunately, I have been unable to find any data relating to the abundance of ladybirds in the Ithaca area, New York State or the surrounding states in 1962, in spite of an extensive literature search, thus the hypothesis of a ladybird population explosion in that area and year must remain speculative. However, the temporal abundance of ladybirds in the juvenile diet of *P. crucifer* [44] also closely matches expectations of aphidophagous ladybird population changes and behavior. Peak dietary abundances occur in early summer directly after ladybird (and frog) breeding. This is followed by a decline (due to ladybird mortality (see [75]) and a slight increase in late summer when ladybirds are very mobile, moving to overwintering sites (e.g., see [76]); more general changes in prey abundances in the diet of *L. pipiens* were also thought to be related to prey life cycles and habits [62]. If coccinellids are taken as prey by frogs and toads in proportion to their occurrence (and apparency) in the habitat, low ladybird numbers in anuran diets probably only indicate their relatively minor faunistic contribution to the potential prey in the habitats of the predators, rather than avoidance related to their chemical defense. 

In most cases, records of ladybird predation probably involve adult beetles (e.g., see [31,50,67]), which have tougher and less easily degraded cuticle that is more easily recognized in stomach samplings. Where predation of larvae is recorded, it can be higher than that of ladybird adults [38], suggesting that much predation of ladybirds (*i.e.*, the soft-bodied larvae) may be missed in dietary inventories. When the species or genera of ladybirds are recorded, they are usually the large and brightly colored members of the aphid-eating tribe Coccinellini (subfamily Coccinellinae). It seems likely that this is because the color patterns make the beetle remains in the guts easier to identify. Thus, from ten years (1915-1924) of analyzing the stomach contents of various North American frogs, Frost [31], records the Coccinellini *Hippodamia parenthesis* (Say), *Hippodamia tredecimpunctata* (L.), *Coccinella transversoguttata* Faldermann, *Adalia bipunctata* (L.), *Coleomegilla maculata lengi* Timberlake (=*Ceratomegilla maculata* (De Geer) and probably *Ceratomegilla fuscilabris* (Mulsant)), as well as from other coccinellid subfamilies *Chilocorus stigma* (Say) (=*C. bivulnerus* Mulsant) and *Hyperaspis undulata* (Say). All of these species exhibit bright coloration [77], presumably warning coloration, and alkaloid defenses have been identified either from these or related species [11,78,79,80]. Other coccinellid prey named in studies include *Cheilomenes lunata* (F.), *Coccinella novemnotata* Herbst, *Coccinella septempunctata* L., *Cycloneda sanguinea* (L.), *Hippodamia convergens* (Guérin-Méneville), *Propylea quatuordecimpunctata* (L.), *Anatis* sp., *Psyllobora* sp., *Scymnus canariensis* Wollaston, *Scymnus cercyonides* Wollaston and *Scymnus trepidulus* Weise [30,38,42,43,49,50,58,66,67], all except the last two of which are apparently warningly colored [43,49,77,81] and known or likely to have alkaloid chemical defenses (see [9,11,12,78,79]). 

### 2.2. Predation by Caudata and Gymnophiona

The low level of predation of ladybirds by newts and salamanders in the comparison (see Section 2, above) was borne out by a wider literature search, which produced only three papers (including the original one) which documented Caudata preying on ladybirds. Two papers relate to the same populations (Jiului National Park, Romania) of same species, the polyphagous European fire salamander, *Salamandra salamandra* (L.) (Salamandridae): five individuals of a total of 177 (2.8%) each ate a single ladybird [82,83]. The third paper recorded two species, both plethodontids, eating ladybirds in a study of five different North American newts and salamanders from the same forest habitat: one of 200 (0.5%) red-backed salamanders, *Plethodon cinereus* (Green), ate a single ladybird adult, and two of 74 (2.7%) adult northern dusky salamanders, *Desmognathus fuscus fuscus* (Green), also ate single adult ladybirds [84].

As already pointed out, it is probably the habitats of salamanders and newts that are responsible for their limited consumption of ladybirds. Many salamanders seem to readily eat other beetles, including members of other chemically defended families, such the soil and litter dwelling Staphylinidae [85,86], as part of a generalist diet (e.g., [84,87,88]). Avoidance of ladybird prey on grounds of their chemical defenses therefore seems unlikely, although it cannot be ruled out. 

The same considerations also apply to the third amphibian order, the caecilians (Gymnophiona), for which no records of ladybird predation have been found. Caecilians generally lead either aquatic or subterranean lives, neither of which is likely to bring them into contact with ladybirds, although it should be noted that ladybirds sometimes spend periods of dormancy underground [76]. At present the very limited data on caecilian diets makes it difficult to rule out caecilian predation of ladybirds, although it seems probable that it is unlikely, or at best very unusual.

## 3. The Acceptability of Ladybird Prey to Amphibians in Captivity

It is clear from gut analyses that coccinellids are regular natural prey for anurans at least. This might be considered surprising given the powerful chemical defenses of ladybirds, which might be assumed to deter amphibians from eating them. Unfortunately, we have very little information on how amphibians react to ladybirds on encountering them. The only published trial of which this author is aware is that of Frazer and Rothschild [89], who tested the common toad (*Bufo bufo* (L.): Bufonidae) with ladybirds as part of more broadly targeted feeding experiments on insect chemical defense. However, they do not comment specifically on these feeding tests, only summarizing ladybirds as overall highly unacceptable for the wide diversity of predators tested. 

Nonetheless, in the course of keeping pet amphibians I have myself observed three cases of predation of ladybirds when these insects were accidentally mixed in with other sweep-netted insects collected as food for anurans. They all involved North American species, from Kentucky (USA). The first case involved a Cope’s grey treefrog (*Hyla chrysoscelis* Cope) eating an adult *Cycloneda munda* (Say). In the other two cases, metamorph American toads (*Anaxyrus americanus* (Holbrook)) ate, in one case an adult *Coleomegilla maculata lengi* and in the other an adult *Hippodamia parenthesis*. All three ladybird species possess alkaloid chemical defenses [12,80,90]. In none of the three cases did the amphibians suffer any obvious subsequent detrimental effects.

It should be emphasized that these were not experiments, but casual observations. However they are striking for a number of reasons. First, because it was thought that they might be harmful, ladybird were typically removed from sweep-netted food: these three cases constituted rare occasions when ladybirds accidentally “got through” into the amphibian cages. They are thus not isolated examples of amphibian predation drawn from a much wider set of encounters where no such predation occurred (although I cannot exclude the possibility that this might occasionally have happened). Second, because the ladybirds were provided among sweep-netted insects, a large number of more palatable prey were simultaneously available to the amphibians, yet the ladybirds were still eaten. Third, the examples involving metamorph *A. americanus* are particularly noteworthy, because the insects were a considerable proportion of the size of the tiny toads. This means that the dose of alkaloid received by these metamorphs was very high. 

## 4. Why Do Amphibians Eat Ladybirds and Why Do They Suffer So Few Ill Effects?

From the evidence given in the previous sections we can make certain generalizations about ladybird-eating in amphibians. It appears that generally the habit is much more frequent in anurans than in the other two groups. This is unsurprising: as already noted the behavior and habitats occupied by anurans mean that they are more likely to come into contact with ladybirds in the first place. As most observations relate to anurans, the discussion that follows is therefore largely framed in terms of the Anura, although it can probably be extended to other amphibian groups. It can also be similarly framed for non-ladybird chemically defended prey: perusal of many of the papers cited here quickly reveals that ladybirds are not the only chemically defended prey occurring in amphibian diets.

Within the Anura there are certainly taxonomic differences in diet—some anurans will live in habitats that mean that they rarely come into contact with ladybirds; there may also be species that genuinely do avoid eating ladybirds or reject them when they do encounter them. But the overall impression is that ladybirds are usually consumed when encountered, as part of a relatively unspecific invertebrate diet. This is supported by the observations for Ithaca in 1962, discussed in Section 2.1. Furthermore, the overall impression is that ladybirds are rarely, if ever, harmful to amphibians when eaten, in contrast to their effects on some other putative ladybird predators (e.g., [91,92]). This poses two questions: first, why are ladybirds taken as prey and not selected out and second, why are they not (more) harmful to the amphibian predators that eat them? 

### 4.1. Why Do Amphibians Not Select Out Ladybirds When Feeding?

Anurans use primarily prey movement to detect their food (e.g., [42,93,94,95,96]). This would tend to indicate that moving ladybirds would be unselectively attacked and eaten. However, frogs can also see and distinguish colors [97], use smell to distinguish between prey [98] and learn on the basis of such cues [99]. All these observations suggest that the visual and olfactory warning signals of ladybirds might act as deterrents to anuran predators. However, experiments on aposematic and olfactory interactions with movement clearly demonstrate that movement remains the primary cue. While frogs attacked grouped moving aposematic prey with a slight delay compared to moving non-aposematic prey, non-moving prey were never attacked; furthermore solitary moving aposematic prey were not attacked significantly less than moving, non-aposematic prey [100]. Odor only acted as a deterrent in slow moving prey, not fast moving [101]. 

The results suggest that ladybirds would gain little protection from anurans/amphibians with the warning signals they possess, because of their behavior. During their active season they typically walk quickly over plants looking for food and mates and this rapid movement would outweigh any deterrent signals. Indeed, because of the strong contrast they present relative to the plants on which they forage, detection of ladybird movement compared to movement of cryptic prey might be easier for anuran and other amphibian predators. Ladybirds might benefit from warning signals against amphibians when overwintering or aestivating, but at these times, which are generally cold or very hot and dry, amphibians are less likely to be active. 

### 4.2. Why Are Ladybirds Not (More) Harmful to the Amphibian Predators That Eat Them? Amphibian Resistance to Ladybird Prey Chemical Defenses

There are three overlapping possible explanations to explain the apparent immunity of amphibians to the toxic effects of ladybird alkaloids. First chemically defended prey can be distasteful, but not toxic. This has been documented for ladybirds in relation to bird predators, but is unlikely to be true in all cases: for example, although the ladybird *Adalia bipunctata* is distasteful but not toxic to nesting blue tits (*Cyanistes* (=*Parus*)*caeruleus* (L.)), *Coccinella septempunctata* is toxic [91]. Certainly a degree of toxicity seems to be a widespread characteristic of ladybird defensive alkaloids [102]. 

A second possibility is that although the alkaloids of the ladybirds consumed are toxic, amphibians generally do not consume a sufficient amount that they are harmful. In some cases this is probably true, although even on the basis of 1 or 2% regularly occurring in gut analyses of a particular amphibian species, this would over time add up to a substantial lifetime alkaloid intake per individual predator, with possible longer term effects. Furthermore, consumption of large numbers of ladybirds is documented in some anurans, but still no harmful effects have been recorded. The consumption of whole adult ladybirds by the juvenile *Anaxyrus americanus* described above (see Section 3) also exposed the amphibians to a substantial alkaloid dose, yet in spite of this, the small toads exhibited no adverse effects. The evidence relating to ladybirds, as well as from other chemically defended prey taxa, suggests that Anura, and probably other amphibians, are at least to some extent resistant to any toxic effects of chemically defended prey that they consume. This is the third and most probable explanation for the lack of harm to amphibians from ladybird alkaloids. Because amphibians are relatively non-specific in the prey they eat as a consequence of their movement-focused mode of hunting, they consume a large number of potentially toxic prey. They have therefore likely evolved physiological means of neutralizing a broad range of potentially harmful defensive chemicals including ladybird alkaloids. This resistance to toxic chemicals ultimately can explain the success of amphibians in preying on ladybirds.

Resistance to the effects of ladybird alkaloids is apparent in other non-selective predators. Majerus [18,81] has pointed out that birds that feed on the wing must be resistant to the toxic effects of ladybirds, because their mode of hunting precludes them selecting their prey. Similarly the web spinning spider *Araneus diadematus* Clerck readily feeds on ladybirds and suffers no obvious ill effects when it does: in this case, the spider’s inability to select out chemically defended prey arises due to its sit-and-wait mode of “hunting”, in a web [20]. Amphibians appear to constitute another example where the way prey are caught leads to limited selectivity and thus the evolution of chemical resistance.

Alkaloid resistance is also already well known for some groups of Anura. A number of lineages of anurans are known to sequester alkaloids from prey for use in their own chemical defenses [103,104]. The known total number of alkaloids used by anurans in this way currently numbers in excess of 800 [105]. Alkaloids shared by ladybirds, and possibly derived from eating them, have been reported from three anuran groups including the Bufonidae [105], a family here recorded frequently preying on ladybirds. Clearly anurans that sequester alkaloids are resistant to their effects, and the sheer number of alkaloids involved suggests a broad range alkaloid resistance, evidently including ladybird alkaloids. Perhaps the sequestration of alkaloids evolved because of the non-specific, motion-orientated feeding of amphibians and a consequent phylogenetically widespread anuran resistance to defensive chemicals. 

## 5. Conclusions

It is tempting to suggest that because of their non-selective predation and the high abundances that they can sometimes reach, anuran populations might in some habitats consume larger numbers of ladybirds than much better-studied predators, such as birds. Certainly there is no reason to ignore the presence of amphibians in the ever increasing list of generalist predators that can and do consume ladybirds. Nonetheless even the effect of anuran predation on populations of ladybirds is probably rather small, because of the high abundances that ladybirds reach. Most studies aimed at examining the effect of amphibians in agricultural settings have generally concluded that their effect on pest species outweighs any effect on beneficial insects, including ladybirds (e.g., [50,106,107]). Indeed in Japanese soybean fields, aphids made up 67.25% of the diet of the Japanese treefrog *Hyla japonica* Günther [107] suggesting that this species and possibly others might be more important as competitors of ladybirds than as predators. It is perhaps worth noting that generally the capacity of natural enemies to regulate ladybird populations is considered to be poor [108]. 

Nonetheless, further studies of the relationship between amphibians and ladybirds would be worthwhile. Studies of the role of warning coloration and odor in deterring amphibian predation are surprisingly limited. In particular, the issue of whether in natural settings warning coloration would serve to emphasize movement and thus increase predation is worthy of consideration and something well suited to study using ladybirds. Similarly ladybird chemical defenses, which are well-characterized chemically, would provide a good way of testing how amphibians respond to naturally occurring defensive allelochemicals. This work might provide an interesting and contrasting counterpoint to work on the responses of amphibians to anthropogenic xenobiotics, which are considered to play a significant role in amphibian declines [109,110,111]. 

This study suggests that in the literature there is a lot of data available on the potential predators of a variety of insect and other invertebrate groups that is being overlooked. Entomologists inevitably concentrate on the literature directly focused on insects, but an exploration of the literature relating to other groups is likely to be of value, perhaps especially in considering vertebrate predation of insects, which, as for ladybirds, is often poorly characterized. The survey of the herpetological literature described here has produced a considerable amount of information on a trophic relationship with amphibians that has rarely warranted any consideration before. Other similar surveys may draw equally surprising results about putative predator groups that have undeservedly received limited attention. 
